# Segmental Odontomaxillary Dysplasia: Review of the Literature and Case Report

**DOI:** 10.1155/2010/837283

**Published:** 2010-12-14

**Authors:** Riya M. Kuklani, Madhu K. Nair

**Affiliations:** ^1^Oral and Maxillofacial Pathology, Department of Oral and Maxillofacial Diagnostic Sciences, College of Dentistry, University of Florida, Gainesville, FL 32610-0414, USA; ^2^Oral and Maxillofacial Radiology, Colleges of Dentistry and Medicine, University of Florida, P.O. Box 100414, Gainesville, FL 32610-0414, USA

## Abstract

Segmental Odontomaxillary Dysplasia (SOD) is an uncommon, nonhereditary, recently recognized developmental disorder affecting the upper jaw and related dental components. It is a rare condition of uncertain etiology that results in painless unilateral expansion of the posterior dentoalveolar complex, gingival hyperplasia, lack of one or both premolars in the affected area, delayed eruption of adjacent teeth and malformations of the primary molars. Radiographically, the affected bone is thickened and irregular in outline, with coarse trabecular pattern that is vertically oriented resulting in a relatively radiopaque granular appearance. Only a few cases have been reported in the English literature. Considering the rarity of the condition, we report a case of SOD in a pediatric patient who was followed up over a period of over two years. The clinical, radiographic, and histologic features are presented along with a review of the literature.

## 1. Introduction

Hemimaxillofacial dysplasia (HD) was first recognized by Miles et al. in 1987 in a report of two cases [1]. This disorder has similar clinical manifestations as Segmental odontomaxillary dysplasia (SOD) but may demonstrate some degree of variable expressivity, for example, facial hypertrichosis is a variable finding [2]. Danforth et al. in 1990 reported a series of eight cases and named the condition [2]. Packota et al. in 1996 reported most common radiographic features of SOD in a study of twelve additional cases [3]. SOD is an uncommon, nonhereditary developmental anomaly involving the maxilla, gingiva and dentition of the same arch. The prevalence of this condition is not well established since the literature largely consists of case reports. To date, 42 cases have been reported in the English literature. The etiology of SOD is unclear. Reports have suggested a viral or bacterial infection as an initial causative factor [4]. Other studies suggested a local developmental abnormality that originates *in utero*. The diagnosis of SOD is mainly based on clinical and radiographic presentation but may be augmented by histological findings. Clinically SOD is characterized by unilateral enlargement of posterior segment of maxilla, enlargement of gingiva, and ipsilateral dental anomolies. The age of the patient at presentation is variable but the condition is usually discovered during childhood with most common complaints being missing teeth, abnormal spacing, and delayed eruption. This condition is slightly more common in males than females. No tendency for its occurrence in any specific ethnic group has been reported. Histologically, the affected bone consists of immature bone with irregular trabeculae of woven appearance with resting or reversal lines without osteoblastic or osteoclastic rimming [5]. Radiographically, vertically oriented trabeculae of woven bone is usually seen which results in a relatively radiopaque, granular appearance. On the affected side, the maxillary sinus may be small [5].

## 2. Case Report

A four-and-a-half-year-old male was referred to the department of pediatric dentistry by a general dental practitioner for evaluation of a painless left maxillary expansile lesion. The patient was otherwise asymptomatic. The major concern was the delayed eruption of primary and permanent teeth in this quadrant. The patient was otherwise healthy with a noncontributory medical history. On clinical examination, the patient revealed mild facial asymmetry caused by an increase in fullness of the left upper lip and cheek. Ipsilateral erythema with increased facial hair of the skin and a scar in the nasolabial region was also seen. Intraoral examination demonstrated buccolingual expansion of maxillary left arch with gingival overgrowth since the age of 3. The left maxillary primary molars exhibited delayed eruption. The gingiva adjacent to primary canine was thickened and appeared red and edematous. The patient was asymptomatic and his oral hygiene was good. His chief complaint was that of delayed eruption of his primary teeth in the same quadrant, especially in the canine and molar area. His left maxillary alveolus was widened with gingival hyperplasia.

**Figure 1:**
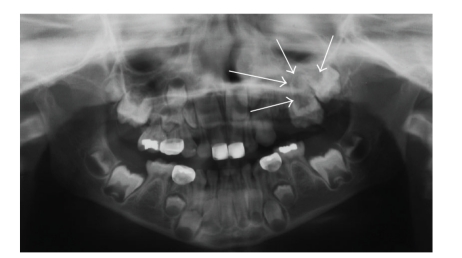
Panoramic radiograph showing lack of/delayed eruption of teeth in the left maxilla.

**Figure 2:**
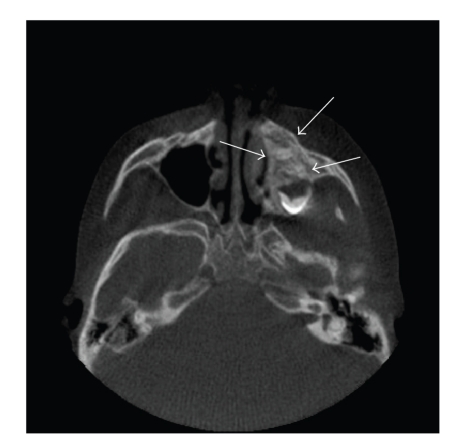
Axial cone beam computed tomography view showing left maxillary alveolar expansile lesion with a dystrophic, ill-defined trabecular pattern.

**Figure 3:**
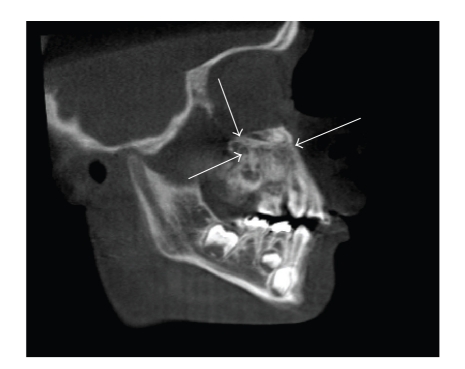
Parasagittal cone beam computed tomography view showing bony changes in the left maxilla with lack of eruption of teeth.

Panoramic radiograph revealed lack of/delayed eruption of teeth with enlargement of the left maxilla (Figure 1). Cone Beam Computed Tomography (CBCT) was obtained to further study the osseous changes in the maxilla. CBCT demonstrated evidence of a left maxillary alveolar expansile lesion with a dystrophic appearance. The cortical plates were well maintained. Trabeculation appeared to be ill defined, and generally oriented along the *y*-axis (Figures 2 and 3). The left maxillary alveolus demonstrated several missing posterior secondary teeth. There was significant expansion of the left maxilla with involvement of the maxillary sinus along the inferior aspect. The nasal fossa was slightly displaced and remodeled on the left side. A clinical impression of fibro- osseous lesion such as fibrous dysplasia or juvenile active ossifying fibroma was made by the surgeon. No lateralization of the air spaces was observed on the CT. Hypoplasia of the left maxillary antrum was observed with the cortical margins being intact. The sinus was partly pneumatized with minimal alveolar extensions. No frank evidence of mucosal thickening or intrasinus fluid was observed. No deviation of the nasal septum was noted. The ostiomeatal complex appeared to be within normal limits. 

**Figure 4:**
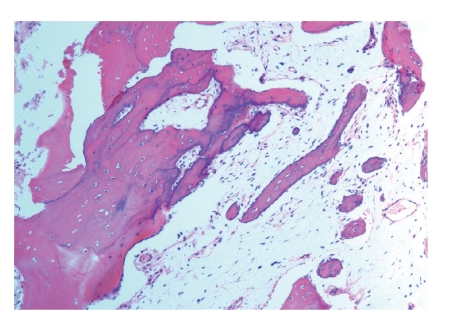
Photomicrograph showing woven bone with numerous basophilic reversal lines with fibrous stroma, while some areas contained peculiar woven bone without fibrous stroma. (magnification ×10).

The patient was subjected to general endotracheal anesthesia and an incisional biopsy of the left maxilla was done. The left maxilla appeared to have a grainy appearance with more normal appearing bone superiorly. The patient healed uneventfully. Histopathologic examination of the decalcified hard tissue revealed mostly woven bone with fibrous stroma, while some areas contained peculiar woven bone without fibrous stroma (Figure 4). Osteoblasts and osteoclasts were not identified. Resting and reversal lines were noted. The histopathologic findings, in the context of clinical findings, led to a diagnosis of SOD. 

Based on the clinical, radiographic, and microscopic features, a diagnosis of SOD was rendered. Reassurance was provided regarding the benign nature of the condition and the patient was placed on periodic recall to monitor the growth and development of the maxillary bone and teeth.

The patient returned after 18 months for a followup evaluation. There was continued evidence of an expansile, hyperattenuating, well-defined lesion occupying the mid-left maxillary dentoalveolus. The floor of the left orbit was intact with no evidence of expansion. The floor of the nasal cavity was unchanged as well. The left maxillary sinus continued to be hypoplastic. No significant increase in the unilateral maxillary swelling was noted. The patient will continue to be monitored on a regular basis.

## 3. Discussion

Hemimaxillofacial dysplasia (HMD) was first recognized in 2 cases by Miles in 1987 which is characterized by unilateral maxillary enlargement, gingival hyperplasia, facial asymmetry, ipsilateral dental abnormalities, unusual radiographic bone pattern, and facial hypertrichosis [1]. Danforth and Melrose reported 8 cases in 1990 in which they termed SOD due to lack of involvement of facial structures in these cases [2]. Packota et al. in 1996 described the criteria for the radiographic diagnosis of SOD as sclerosis of bone with thickened trabeculae, missing premolars with delayed eruption of permanent teeth, vertical orientation of bony trabeculae, spacing between deciduous molars, and a small maxillary sinus on the affected side. Our case features most of the criteria put forth by Packota et al. [3]. The acronym HATS (**H**emimaxillary enlargement, **A**symmetry of the face, **T**eeth abnormalities, **S**kin findings) was introduced in 2004 by Welsch and Stein [6]. They reported two cases with skin lesions. One patient reported having Becker's nevus. All cases reported appeared to represent sporadic occurrence with no inheritance pattern.

The prevalence of this condition is not well established since the literature largely consists of case reports. According to a previous literature review, 27 cases of SOD/HMD had been published from 1987 to 2000 [1–3, 7–10]. 15 additional cases reported since then were retrieved from PubMed database. The number of cases reported in the extant literature still remains variable since not all cases are well documented. We present the most common clinical features, radiologic features, and cutaneous findings (Tables 1 and 2) [1–18] for a total of 43 cases including this case. 

Clinically, SOD usually presents as a non-progressive facial asymmetry, ipsilateral gingivo-dento-alveolar maxillary involvement which can extend from the canine to tuberosity area [10]. Facial cutaneous lesions may or may not be present. Radiographically, SOD shows ill-defined bony sclerosis with thickened and coarse bony trabecular pattern [3]. Histopathology is not very specific for SOD. The gingival thickening shows nonspecific noninflammatory connective tissue hyperplasia. Osseous involvement demonstrates the presence of thick trabeculae of immature woven bone with prominent reversal and resting lines, noninflammatory fibrous stroma with lack of osteoblastic and clastic activity [5]. Other dental defects could include pulp stones and irregular pulpal or dentinal interface. 

SOD may go unrecognized or misdiagnosed because some conditions like fibrous dysplasia and regional odontodysplasia (ROD) shows similarities to SOD, but they can be differentiated from SOD (Table 3). ROD displays segmental involvement with no expansion. In addition, ROD does not demonstrate unilateral enlargement of the gingiva and associated alveolar ridge and usually affects permanent anterior teeth only. Radiographically, hypoplastic dental tissue presents as ghost teeth [5]. In addition, ROD does not demonstrate unilateral enlargement of the gingiva and associated alveolar ridge. Fibrous dysplasia and SOD have similar radiographic features but fibrous dysplasia is not noted for absence of teeth. Although Fibrous Dysplasia (FD) often is associated with a similar clinical expansion of the alveolar ridge, the radiographic and histopathologic features are very different. Upon imaging, craniofacial FD presents as an ill-defined zone of fine trabeculation which contrasts very clearly with the course trabecular pattern noted in SOD. Histopathologically, immature fibrous dysplasia presents with irregular trabeculae of vital woven bone which resembles “Chinese characters”, does not demonstrate numerous reversal lines, and typically reveals significant osteoid rimming.

Treatment of SOD remains unknown as the management protocol in most case reports has not been discussed to date. Once diagnosed, SOD seems to remain stable and may or may not require surgical intervention. So the primary goal of the treatment is to retain the deciduous teeth, thus facilitating eruption of the permanent teeth so that occlusion can be restored [10]. Usually definitive treatment is delayed until after the pubertal growth spurt. Standard of care is close observation with most clinicians performing limited recontouring in cosmetically objectionable cases. The lack of reports in adults suggests the possibility of spontaneous regression with age. In our case, no treatment was advised at this time. But it is important to recognize the existence of the condition and diagnose it using appropriate clinical and radiographic findings. Even though SOD is a rare entity, the radiologist must consider SOD in the differential diagnosis of entities such as monostotic fibrous dysplasia, regional odontodysplasia, hemifacial hyperplasia, and gingival fibromatosis. The diagnosis is made by exclusion. The clinical and radiological features of SOD are well described but the condition is probably underreported due to misdiagnosis. Thus, it is important to recognize this unusual unilateral developmental anomaly.

## 4. Conclusion

SOD is a rare condition. Since it affects children care should be taken to accurately diagnose the condition to reassure the patient and offer the best treatment options. The diagnosis of SOD is primarily based on the clinical and radiographic findings. Treatment may be limited to retaining the primary teeth and enhancing the eruption of the permanent teeth, if possible, to restore occlusion in that quadrant.

## Figures and Tables

**Table 1:** Clinical features of 43 patients with segmental odontodysplasia.

Author	no. of Cases	Facial asymmetry	Gingival thickening	Hypodontia	Maxillary/alveolar thickening	Hypoplastic teeth
Miles et al.	2	2/2	2/2	1/2	2/2	2/2
Danforth et al.	8	3/8	4/8	8/8	5/8	3/8
Packota et al.	1	0	1/1	1/1	1/1	NS
Packota et al.	2–12	NS	NS	11/11	6/11	NS
De Salvo et al.	1	1/1	1/1	1/1	1/1	1/1
Paticoff et al.	2	2/2	2/2	2/2	2/2	2/2
Jones & Ford et al.	1	0/1	1/1	1/1	1/1	NS
Prusack et al.	1	1/1	1/1	1/1	1/1	NS
Velez et al.	2	1/2	2/2	2/2	2/2	2/2
Becktor et al.	4	NS	4/4	4/4	4/4	NS
Drake et al.	1	1/1	1/1	1/1	1/1	NS
Armstrong et al.	2	2/2	2/2	2/2	2/2	1/2
Welsh & Stein et al.	1	1/1	1/1	1/1	1/1	0
Gavalda et al.	1	1/1	1/1	1/1	1/1	1/1
Özpinar et al.	1	0	0	1/1	1/1	0
Koenig et al.	1	1/1	1/1	1/1	1/1	1/1
Porwal et al.	1	1/1	0	1/1	0	0
Yassin et al.	1	1/1	1/1	1/1	1/1	0
Present Case	1	1/1	1/1	1/1	1/1	0

*NS: not stated; 0: absent.

**Table 2:** Radiologic features and cutaneous findings of 43 patients with segmental odontodysplasia [1–18].

Author	# of Cases	Hyper-trichosis	Other cutaneous findings	Vertically Oriented trabeculae	Thickened bone trabeculae	Teeth separated/ displaced	Decreased Maxillary Sinus Size	Root Resorption	Other Radiologic findings
Miles et al.	2	1/2	NS	NS	NS	2/2	NS	0	Bone dense and granular
Danforth et al.	8	0	NS	NS	NS	6/8	1/8	6/8	Mottled/ill-defined opaque
Packota et al.	1	0	Discontinuity of the left vermilion border, depression of cheek	9/12	12/12	10/12	9/12	6/12	—
Packota et al.	2–12	NS	—						
De Salvo et al.	1	0	Hypo pigmented lip	NS	NS	0	1/1	0	Ill defined coarse trabecular pattern
Paticoff et al.	2	2/2	1/2 hairy nevus	NS	NS	NS	NS	NS	Hyper plastic bone
Jones & Ford et al.	1	1/1	Beckers nevus	1/1	1/1	0	NS	1/1	—
Prusack et al.	1	0	—	1/1	1/1	1/1	1/1	1/1	Bone changes extending to zygoma and orbit
Velez et al.	2	1/2	Hyperpigmentation of facial skin	1/2	1/2	0	0	0	Expansile diffuse radioopaque
Becktor et al.	4	0	2/4 Erythema	0	4/4	4/4	3/4	3/4	Sclerosis
Drake et al.	1	0	—	NS	NS	1/1	0	0	Ill defined opacity
Armstrong et al.	2	0	—	0	0	2/2	0	0	Ill defined radio density
Welsh & Stein et al.	1	1/1	Beckers nevus	0	1/1	1/1	0	0	—
Gavalda et al.	1	NS	NS	0	0	0	0	0	—
Özpmar et al.	1	0	—	0	1/1	0	0	0	—
Koenig et al.	1	1/1	Erythema, lipclefting, hyper linear palms, depression of cheek	0	1/1	1/1	NS	NS	Sclerotic, ground glass
Porwal et al.	1	1/1	Hypo pigmented streak	0	0	0	1/1	0	—
Yassin et al.	1	0	Hypo pigmentation of lip, erythema	0	1/1	0	1/1	0	—
Present Case	1	1/1	Erythema, scar in nasolabial region, increased fullness of upper lip	1/1	1/1	0	1/1	0	Nasal fossa displaced and remodeled

*NS: not stated; 0: absent.

**Table 3:** Features to distinguish between SOD, ROD and Fibrous Dysplasia.

Entity	Clinical presentation	Soft tissue enlargement	Missing teeth	Expansion	Radiographic appearance
SOD	Painless, unilateral enlargement of maxillary bone	Yes	Pre-molars one or both	Yes	Course trabecular pattern
ROD	Affects maxillary anterior with failure or delayed eruption of teeth	May be present	Yes	No	Ghost teeth present
Fibrous Dysplasia	Localized painless swelling involving one or more bones	Slow growing painless swelling present	No	Yes	Ground glass radio-opacity or ill-defined zone of fine trabeculation

ROD: Regional odonto-dysplasia, SOD: Segmental odonto-dysplasia.
